# Evocative effects of children's education‐associated genetics on maternal parenting: results from the Norwegian mother, father and child cohort study

**DOI:** 10.1111/jcpp.70025

**Published:** 2025-08-04

**Authors:** Chloe Austerberry, Tetyana Zayats, Angelica Ronald, Elizabeth Corfield, Dinka Smajlagic, Alexandra Havdahl, Ole A. Andreassen, Per Magnus, Pål R. Njølstad, Mona Bekkhus, Pasco Fearon

**Affiliations:** ^1^ Centre for Child, Adolescent and Family Research University of Cambridge Cambridge UK; ^2^ Analytic and Translational Unit Massachusetts General Hospital Boston MA USA; ^3^ Stanley Center for Psychiatric Disorders Broad Institute of MIT and Harvard Cambridge MA USA; ^4^ PROMENTA Research Center, Department of Psychology University of Oslo Oslo Norway; ^5^ Program in Medical and Population Genetics Broad Institute of MIT and Harvard Cambridge MA USA; ^6^ School of Psychology University of Surrey Guildford UK; ^7^ PsychGen Centre for Genetic Epidemiology and Mental Health Norwegian Institute of Public Health Oslo Norway; ^8^ Nic Waals Institute Lovisenberg Diakonale Hospital Oslo Norway; ^9^ Population Health Sciences, Bristol Medical School University of Bristol Bristol UK; ^10^ Division of Mental Health and Addiction, NORMENT Centre Oslo University Hospital and University of Oslo Oslo Norway; ^11^ KG Jebsen Centre for Neurodevelopmental Disorders University of Oslo Oslo Norway; ^12^ Centre for Fertility and Health Norwegian Institute of Public Health Oslo Norway; ^13^ Department of Clinical Science University of Bergen Bergen Norway; ^14^ Children and Youth Hospital Haukeland University Hospital Bergen Norway

**Keywords:** Behavioral genetics, educational attainment, parenting, language, structural equation modeling

## Abstract

**Background:**

It has long been hypothesized that increasing heritability with age of cognitive and educational performance is partly attributable to evocative gene–environment correlation. However, this hypothesis has not been widely tested.

**Methods:**

We addressed this gap by examining whether children's education polygenic scores (PGS_edu_ ) were associated with maternal self‐reported positive and literacy‐focused parenting when children were 5 years old, and if evoked parenting differences mediated genetic effects on children's educational outcomes (mother‐reported at 6–8 years of age), while controlling for parental PGS_edu_ . We also investigated whether maternal reports of children's language at 5 years old were associated with parenting and mediated genetic effects on educational performance. These questions were addressed in a sample of 83,627 parent‐offspring trios from the Norwegian Mother, Father and Child Cohort Study, a longitudinal population‐based pregnancy cohort.

**Results:**

Children's PGS_edu_ were significantly associated with maternal literacy‐focused (β = .03, 95% CI [0.01, 0.05], *p* = .021) but not positive parenting (β = 0.01, 95% CI [−0.02, 0.05], *p* = .410), and literacy‐focused parenting significantly mediated the effects of children's PGS_edu_ on their educational performance (β = 0.01, 95% CI [1 × 10^−3^, 0.01], *p* = .023). Children's language was associated with maternal parenting and mediated the effects of children's PGS_edu_ on their educational performance (β = 0.01, 95% CI [3 × 10^−3^, 0.02], *p* = .002).

**Conclusions:**

These findings support our hypotheses and suggest early language and parenting may be mechanisms implicated in the pathways from children's genetics to their educational outcomes.

## Introduction

Cognitive and educational abilities are important assets and strong predictors of health and longevity (Deary, Weiss, & Batty, 2010; Hummer & Hernandez, 2013; Kosik et al., 2018). Their heritability is well established (Okbay et al., 2022; Silventoinen et al., 2020) and has been found to increase from approximately 20%–50% in childhood to 50%–80% in adulthood (Bouchard Jr. & McGue, 1981; Haworth et al., 2010; Kovas et al., 2013), ostensibly suggesting the influence of environments diminishes with age. A plausible alternative, investigated in this study, is that increasing heritability estimates mask key environmental processes that mediate genetic influences through mechanisms of gene–environment correlation (*r*GE), amplifying genetic effects over time (Dickens & Flynn, 2001; Plomin, DeFries, & Loehlin, 1977; Scarr & McCartney, 1983).

While many recent studies have investigated the indirect influence of *parents'* genes on children's educational outcomes via environmental mechanisms (Kong et al., 2018; Wang et al., 2021), far less research has examined the mediated influence of *children's* genes on their educational outcomes via environmental mechanisms such as evoked differences in the social environment. Evocative *r*GE occurs when individuals' genetically influenced characteristics systematically evoke differences in their environment, potentially mediating genetic effects (Bell, 1968; Shaw & Bell, 1993). As these environmental effects are correlated with genetic differences, their significance can be obscured by focusing purely on global heritability estimates.

Phenotypic research indicates children's characteristics may elicit responses from their caregivers (Bell, 1968; Blair, Raver, Berry, & Family Life Project Investigators, 2014; Hipwell et al., 2008; Lugo‐Gil & Tamis‐LeMonda, 2008; Pardini, Fite, & Burke, 2008). Additionally, behavioral genetics findings suggest children's genetic differences evoke differences in parenting (Klahr et al., 2017; Plomin & Bergeman, 1991), including in early and middle childhood (Boivin et al., 2005; Elam et al., 2014; Fearon et al., 2015; Harold et al., 2013; Klahr et al., 2017; Knafo & Plomin, 2006). Crucially, only a small subset of this literature focuses on cognitive and educational abilities. One multivariate twin study found positive associations between cognition at age 2 years and cognitively stimulating parenting at 4 years were almost entirely genetically mediated (Tucker‐Drob & Harden, 2012). One parent‐offspring adoption study found genetic factors underlying adoptee intellect were positively associated with adoptive parent positive parenting at ages 6 and 7 years (Austerberry et al., 2024). Two previous studies have used education polygenic scores (PGS_edu_) to examine evocative *r*GE in educational development: Krapohl et al. (2017) found that children's PGS_edu_ were associated with early caregiving. However, as they did not control for parental genetics, it was not possible to rule out passive *r*GE (which occurs when parents' genes influence their children's environments, and children passively inherit some of these genes, inducing a spurious gene–environment association). Wertz et al. (2020) found children's PGS_edu_ were positively associated with positive and cognitively stimulating maternal parenting and negatively associated with household chaos, after controlling for mothers' PGS_edu_. To our knowledge, evocative effects of children's PGS_edu_ have never been examined while controlling for the genetics of both parents. Nor have any previous analyses examined whether evoked parenting mediates the effects of PGS_edu_ on children's outcomes. We aimed to examine both gaps in the literature.

Studies have suggested language may be a key early manifestation of genetic influences on education (Austerberry et al., 2022; Verhoef, Shapland, Fisher, Dale, & St Pourcain, 2021) and may evoke differences in dimensions of parenting that support cognitive development (Tucker‐Drob & Harden, 2012). Thus, we also aimed to investigate the role of early language.

We tested the following three preregistered hypotheses: (1) children's PGS_edu_ would be positively associated with maternal positive and cognitively stimulating (literacy‐focused) parenting, after controlling for parents' PGS_edu_; (2) maternal parenting would mediate the association between children's PGS_edu_ and educational performance; (3) children's language would mediate PGS_edu_ effects on educational performance and be associated with parenting.

## Methods

### Sample

Data were from the Norwegian Mother, Father, and Child Cohort Study (MoBa), a population‐based pregnancy cohort conducted by the Norwegian Institute of Public Health (Magnus et al., 2006, 2016). Pregnant women were recruited from across Norway from 1999 to 2008. The women consented to participation in 41% of pregnancies (*N* = 112,908 recruited pregnancies). The cohort now includes 114,500 children, 95,200 mothers, and 75,200 fathers. The current study is based on version 12 of the quality‐assured data files released for research in January 2019. The establishment of MoBa and initial data collection was based on a license from the Norwegian Data Protection Agency and approval from the Regional Committees for Medical and Health Research Ethics (REK). The MoBa cohort is based on regulations of the Norwegian Health Registry Act. The current study was approved by the REK (21076). Blood samples were collected from both parents during pregnancy and from children (umbilical cord) at birth (Paltiel et al., 2014). Further information on recruitment and data collection is reported in published cohort profiles (Magnus et al., 2006, 2016). Protocols, including consent forms and questionnaires, are published elsewhere (Norwegian Institute of Public Health, 2019).

### Genotyping, quality control, and imputation

Genotyping, quality control, and imputation of MoBa genetic data is described elsewhere (Corfield et al., 2022). In short, following standard pre‐imputation quality control, the genotypes of individuals of European descent were imputed using the Haplotype Reference Consortium (HRC) panel (McCarthy et al., 2016).

Using the data released by the MoBaPsychgen group (Corfield et al., 2022), we created a subsample of unrelated parent‐offspring trios with at least one member genotyped (161,566 individuals [63,032 children, 58,778 mothers, 39,756 fathers]). The steps performed to create this subsample are outlined in Appendix S1.

### Measures

#### Education polygenic scores

Genetic propensity for educational attainment was quantified using education polygenic scores (PGS_edu_). We constructed the PGS_edu_ using PRS‐CS (Ge, Chen, Ni, Feng, & Smoller, 2019) and summary statistics (not including 23andMe) from the Okbay et al. (2022) education genome‐wide association study (GWAS; see Appendix S2).

#### Positive parenting

Using confirmatory factor analysis (CFA), we constructed a latent variable using six maternal self‐report items when children were 5 years old from the ‘Positive Parenting’ subscale of the Alabama Parenting Questionnaire (Frick, 1991), a valid and reliable measure (Essau, Sasagawa, & Frick, 2006; Frick, Christian, & Wootton, 1999; Shelton, Frick, & Wootton, 1996). Items were scored on a 5‐point Likert (‘1‐Never’ to ‘5‐Always’). Higher scores indicated more positive parenting. Reliability was acceptable (α = .78) in the sample. As with the below measures of literacy‐focused parenting and language, the positive parenting measure was selected based on an exploratory factor analysis (EFA) conducted prior to hypothesis testing (see Appendix S3 and Tables S1–S3).

#### Literacy‐focused parenting

We created a total score by averaging two maternal self‐report items measuring parental support with literacy at age 5 years, selected from the Early Language in Victoria Study (Prior, Bavin, & Ong, 2011): ‘During a typical week, how often do you (1) teach your child how to print letters and words?’; (2) ‘help your child read letters and sounds?’ Both were scored on a 5‐point Likert (‘1‐Never’ to ‘5‐Very Often’). The two items were highly correlated (*r* = .68, *p* < .001).

#### Language

We constructed a latent variable with 13 indicators from the mother‐reported Speech and Language Assessment Scale (SLAS) at age 5 years. The SLAS is a valid and reliable measure capturing children's articulation, semantics, vocabulary, sentence construction, and conversational skills compared with peers (Hadley & Rice, 1993; Rice, Wilcox, Liebhaber, & Hadley, 1989). All items were scored on a 5‐point Likert (‘1‐Very much lower’ to ‘5‐Very much higher’). Reliability was excellent (α = .96).

#### Educational performance

We constructed a latent variable from three items, reported by mothers at 8 years, on their child's national exam performance on: (1) Reading in first grade, (2) Reading in second grade, (3) Arithmetic in second grade. Items were scored on a 3‐point Likert: ‘1‐Teacher is concerned’, ‘2‐Must work more but teacher is not concerned’, ‘3‐Has mastered subject well’. Reliability was acceptable (α = .77).

### Statistical analyses

The analyses were preregistered (Austerberry et al., 2022). In the main analyses, we included all unrelated parent‐offspring trios with post‐QC genetic data on at least one trio member (83,627 trios; Table 1). Hypotheses were tested in lavaan version 0.6–10 (Rosseel, 2012), R 4.0.3 (R Core Team, 2020), in structural equation models (SEMs) outlined in Appendix S4. *p* < .05 was the threshold for statistical significance. Missing data were handled using full information maximum likelihood (FIML; Appendix S5). Mediation was estimated using 1,000 bootstraps (Bollen & Stine, 1990). Model fit was considered adequate if the standardized root mean square residual (SRMR) was <.09 and the root mean square error of association (RMSEA) < .06 (Hu & Bentler, 1999). We calculated the variance inflation factors (VIFs) in a multiple regression with the three PGS_edu_ as predictors. VIF >4 was the multicollinearity threshold.

**Table 1:** Count and percentage for each condition of complete or incomplete genetic data

PGS_child_	PGS_mother_	PGS_father_	*n*	%
✓	×	×	11,132	13.31
×	✓	×	9,713	11.61
×	×	✓	4,727	5.65
✓	✓	×	23,026	27.53
✓	×	✓	8,990	10.75
×	✓	✓	6,155	7.36
✓	✓	✓	19,884	23.78

PGS, years of education polygenic score. ✓ = data available. × = missing data. Total *n* = 83,627 trios.

We conducted two sensitivity analyses, re‐running the analyses on (1) a subsample of 19,884 trios with post‐QC genetic data for all three members; (2) all unrelated trios with some post‐QC genetic or phenotypic data (96,577 trios).

## Results

### Descriptive statistics and bivariate associations

Table 2 presents the descriptive statistics. Mean maternal self‐reports of positive parenting ranged from 4.19 to 4.78 (on a 1–5 scale), indicating mothers tended to view themselves as between “often” (4) and “always” (5) positive. The mean score of how often mothers helped their child to read or write was 3.40 (on a 1–5 Likert), demonstrating they tended to rate themselves as between “sometimes” (3) and “often” (4) engaging in literacy‐focused parenting. Mean language ratings ranged from 3.43 to 3.72 (on a 1–5 Likert), indicating mothers tended to rate children as slightly above “typical for their age” (3). Mean reading and math scores ranged from 2.63 to 2.81 (on a 1–3 Likert), indicating mothers tended to rate children as between “must work more but teacher is not concerned” (2) and “has mastered subject well” (3).

**Table 2:** Means, standard deviations and bivariate correlations between genetic and phenotypic variables

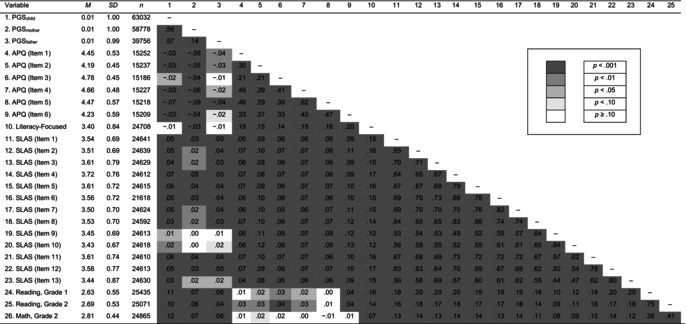

APQ, Alabama Parenting Questionnaire; Literacy‐Focused, literacy‐focused parenting; PGS, years of education polygenic score; SLAS, Speech and Language Assessment Scale.

**Figure 1:**
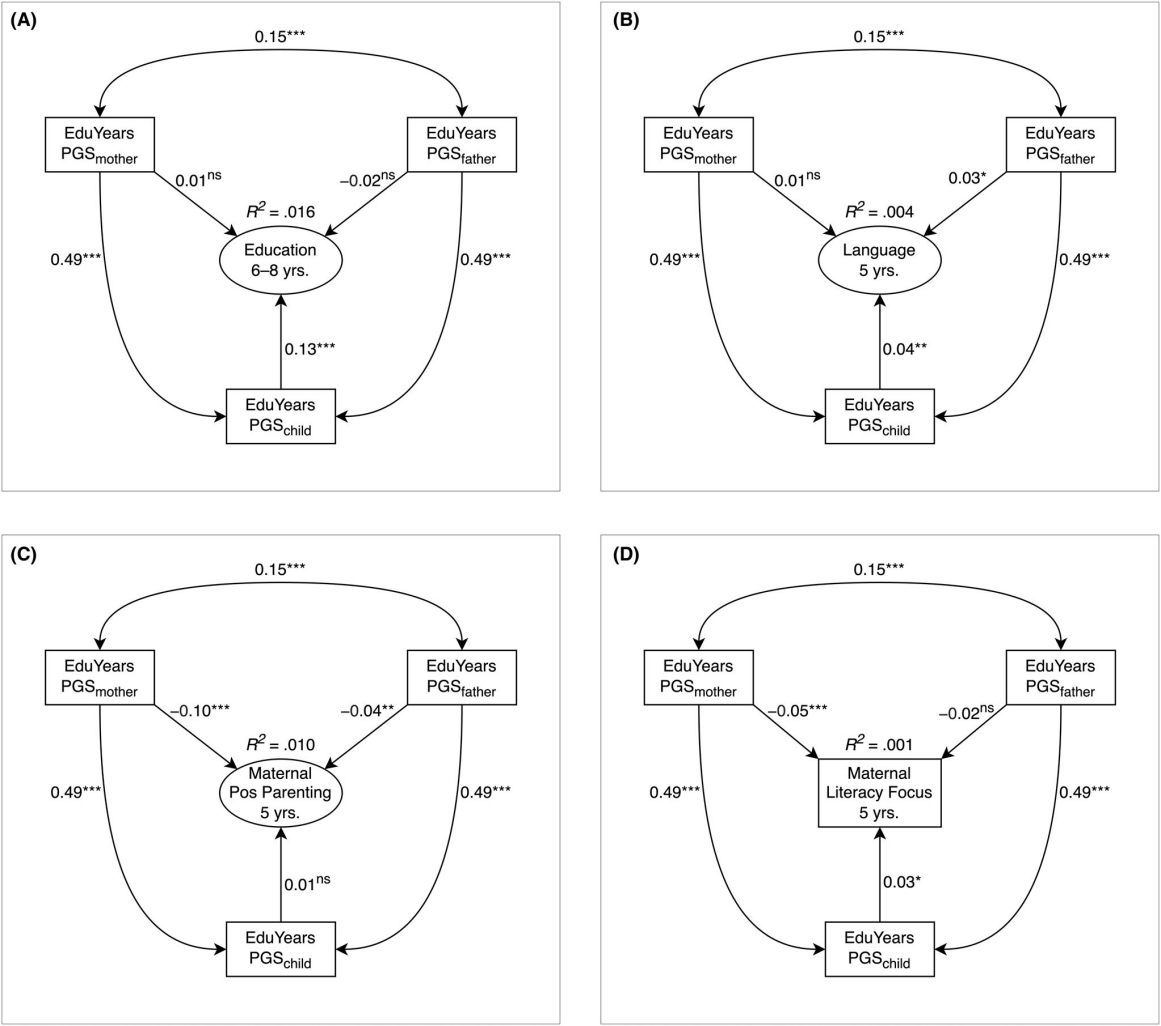
Preliminary trio models examining associations between polygenic scores and phenotypic variables. PGS, years of education polygenic score. Pos = Positive. (A) Fit: χ^2^(6) = 137.96, *p* < .001, comparative fit index (CFI) = .99, root mean square error of approximation (RMSEA) = .02, standardized root mean square residual (SRMR) = .02. (B) Fit: χ^2^(101) = 40,702.01, *p* < .001, CFI = .88, RMSEA = .07, SRMR = .04. (C) Fit: χ^2^(24) = 1,440.33, *p* < .001, CFI = .97, RMSEA = .03, SRMR = .03. (D) Fit: χ^2^(6) = 29,077.99, *p* < .001, CFI = 1.00, RMSEA = .00, SRMR = .00. Standardized estimates are reported. ^ns^ *p* ≥ .10; *p < .05; **p < .01; ***p < .001

Bivariate structural equation models (SEMs) examining the effects of each PGS_edu_ are reported in Table 3. Trio SEMs modelling the effects of the PGS_edu_ together are reported in Figure 1.

**Table 3:** Bivariate structural equation models examining associations between each polygenic score individually and phenotypic variables

Hypothesized association	β	CI_95% lower_	CI_95% upper_	*SE*	*p* value	*R* ^2^
Child PGS → Educational Performance 6–8 years	0.13	0.11	0.14	0.01	<.001	.02
Mother PGS → Educational Performance 6–8 years	0.08	0.06	0.09	0.01	<.001	.01
Father PGS → Educational Performance 6–8 years	0.05	0.04	0.07	0.01	<.001	3 × 10^−3^
Child PGS → Language 5 years	0.06	0.04	0.07	0.01	<.001	3 × 10^−3^
Mother PGS → Language 5 years	0.03	0.02	0.05	0.01	<.001	1 × 10^−3^
Father PGS → Language 5 years	0.05	0.03	0.06	0.01	<.001	2 × 10^−3^
Child PGS → Maternal Positive Parenting 5 years	−0.06	−0.08	−0.05	0.01	<.001	4 × 10^−3^
Mother PGS → Maternal Positive Parenting 5 years	−0.10	−0.12	−0.08	0.01	<.001	.01
Father PGS → Maternal Positive Parenting 5 years	−0.05	−0.07	−0.02	0.01	<.001	2 × 10^−3^
Child PGS → Maternal Literacy‐Focused Parenting 5 years	−0.01	−0.02	4 × 10^−3^	0.01	.172	5 × 10^−5^
Mother PGS → Maternal Literacy‐Focused Parenting 5 years	−0.04	−0.05	−0.02	0.01	<.001	1 × 10^−3^
Father PGS → Maternal Literacy‐Focused Parenting 5 years	−0.01	−0.03	0.01	0.01	.285	6 × 10^−5^

PGS, years of education polygenic score.

### Trio model of educational performance

Although in the bivariate associations (Table 3) all PGS_edu_ were significantly associated with children's educational performance, in the trio SEM (Figure 1A), only the child PGS_edu_ was significantly associated with child education (PGS_child_: β = 0.13, 95% CI [0.11, 0.16], *p* < .001; PGS_mother_: β = 0.01, 95% CI [−0.01, 0.03], *p* = .373; PGS_father_: β = −0.02, 95% CI [−0.04, 0.01], *p* = .145). Mother and father PGS_edu_ were associated (β = 0.15, 95% CI [0.14, 0.16], *p* < .001), indicating assortative mating.

### Trio model of language

Although in the bivariate models (Table 3) all PGS_edu_ were significantly associated with children's language, in the trio SEM (Figure 1B) only child and father PGS_edu_ were significantly associated with child language (PGS_child_: β = 0.04, 95% CI [0.01, 0.06], *p* = .003; PGS_mother_: β = 0.01, 95% CI [−0.01, 0.03], *p* = .281; PGS_father_: β = 0.03, 95% CI [3 × 10^−3^, 0.05], *p* = .026).

### Test of hypothesis 1: Evocative effects of Children's polygenic scores on parenting

#### Trio model of positive parenting

There was little evidence of evocative effects on maternal positive parenting. While in the bivariate models (Table 3) children's PGS_edu_ were significantly (unexpectedly, negatively) associated with positive parenting, in the trio model controlling for parent PGS_edu_ (Figure 1C) children's PGS_edu_ were not significantly associated with maternal positive parenting (β = 0.01, 95% CI [−0.02, 0.05], *p* = .410). As in the bivariate models (Table 3), parent PGS_edu_ were significantly (unexpectedly, negatively) associated with maternal positive parenting (PGS_mother_: β = −0.10, 95% CI [−0.12, −0.07], *p* < .001; PGS_father_: β = −0.04, 95% CI [−0.07, −0.01], *p* = .009).

#### Trio model of literacy‐focused maternal parenting

There was stronger evidence of possible evocative effects on maternal literacy‐focused parenting. Unlike in the bivariate model (Table 3), in the trio model (Figure 1D) children's PGS_edu_ were significantly associated with maternal cognitive stimulation (β = 0.03, 95% CI [4 × 10^−3^, 0.05], *p* = .021). As in the bivariate models (Table 3), the effect of mothers' but not fathers' PGS_edu_ on maternal cognitive stimulation in the trio model was significant (PGS_mother_: β = −0.05, 95% CI [−0.07, −0.03], *p* < .001; PGS_father_: β = −0.02, 95% CI [−0.04, 4 × 10^−3^], *p* = .099). Unexpectedly, this significant association was negative.

### Test of hypothesis 2: Polygenic score effects on education mediated via parenting

**Figure 2:**
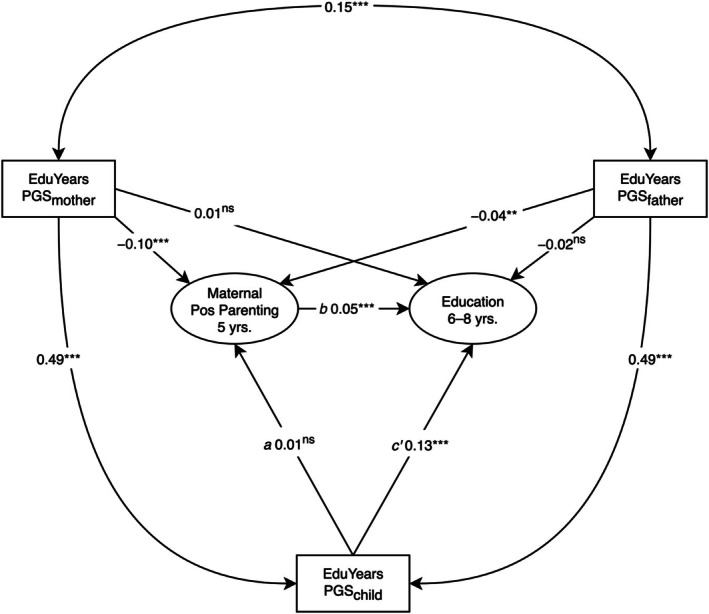
Longitudinal structural equation model testing hypothesis 2: mediated effect of children's polygenic scores on educational performance via maternal positive parenting. PGS, years of education polygenic score. Pos = Positive. Model fit: χ^2^(47) = 1611.36, *p* < .001, comparative fit index = .98, root mean square error of approximation = .02, standardized root mean square residual = .02, standardized estimates reported. ^ns^ *p* ≥ .1; **p < .01; ***p < .001

Although maternal positive parenting was significantly associated with children's educational performance (Figure 2; β = 0.05, 95% CI [0.02, 0.07], *p* < .001), it did not significantly mediate the effect of children's PGS_edu_ on their educational performance (a*b: β = 1 × 10^−3^, 95% CI [−1 × 10^−3^, 2 × 10^−3^], *p* = .406). The model explained 2% of the variance in educational performance.

**Figure 3:**
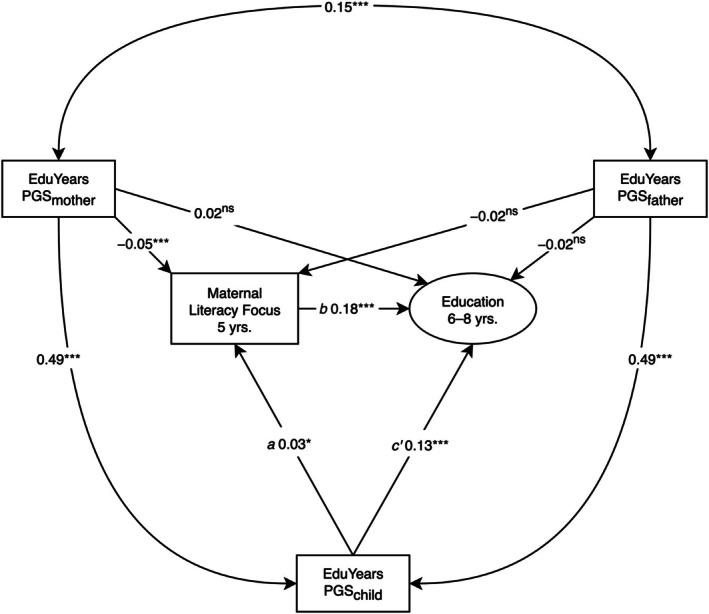
Longitudinal structural equation model testing hypothesis 2: mediated effect of children's polygenic scores on educational performance via maternal literacy‐focused parenting. PGS, years of education polygenic score. Model fit: χ^2^(8) = 163.85, *p* < .001, comparative fit index = 1.00, root mean square error of approximation = .02, standardized root mean square residual = .01, standardized estimates reported. ^ns^ *p* ≥ .10; ***p < .001

Maternal literacy‐focused parenting at 5 years was significantly associated with children's educational performance (Figure 3; β = 0.17, 95% CI [0.16, 0.19], *p* < .001) and significantly mediated the effect of children's PGS_edu_ on their educational performance (a*b: β = 0.01, 95% CI [1 × 10^−3^, 0.01], *p* = .023). The model explained 5% of the variance in educational performance.

### Test of hypothesis 3: Polygenic score effects on education mediated via language

**Figure 4:**
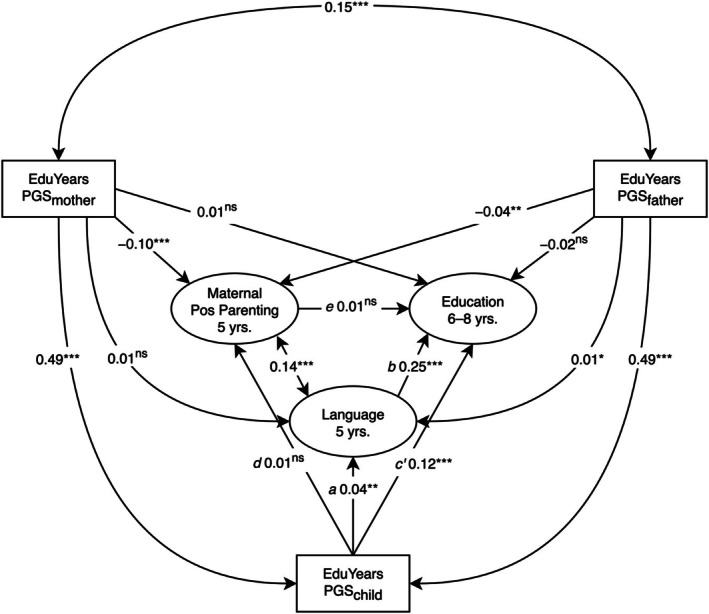
Longitudinal structural equation model testing hypothesis 3: mediated effect of children's polygenic scores on educational performance via language. PGS, years of education polygenic score. Pos = Positive. Model fit: χ^2^(263) = 42,945.52, *p* < .001, comparative fit index = .89, root mean square error of approximation = .04, standardized root mean square residual = .04, standardized estimates reported. ^ns^ *p* ≥ .10; **p < .01; ***p < .001

**Figure 5:**
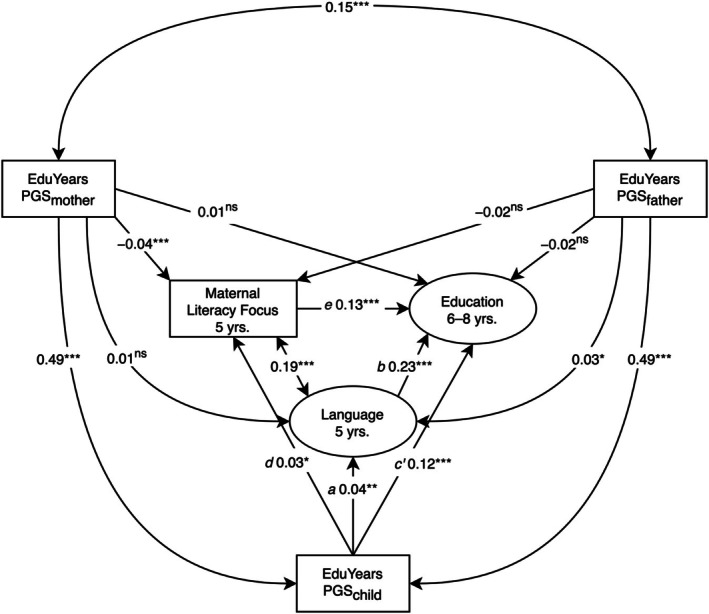
Longitudinal Structural Equation Model Testing Hypothesis 3: Mediated Effect of Children's Polygenic Scores on Educational Performance via Language. PGS, years of education polygenic score. Model fit: χ^2^(159) = 41,231.90, *p* < .001, comparative fit index = .89, root mean square error of approximation = .05, standardized root mean square residual = .04, standardized estimates reported. ^ns^ *p* ≥ .10; *p < .05; **p < .01; ***p < .001

Language significantly mediated the effects of children's PGS_edu_ on educational performance in the positive parenting model (Figure 4; a*b: β = 0.01, 95% CI [3 × 10^−3^, 0.02], *p* = .002) and cognitive stimulation model (Figure 5; a*b: β = 0.01, 95% CI [3 × 10^−3^, 0.01], *p* = .002). Language was significantly associated with maternal positive parenting (Figure 4; β = 0.14, 95% CI [0.12, 0.15], *p* < .001) and cognitive stimulation (Figure 5; β = 0.19, 95% CI [0.18, 0.20], *p* < .001). The positive parenting (Figure 4) and cognitive stimulation (Figure 5) SEMs explained 8% and 10% of the variance in educational performance, respectively.

### Multicollinearity

The variance inflation factors (VIF) for the trio polygenic scores were <4 (PGS_child_, 2.24; PGS_mother_, 1.55; PGS_father_, 1.58). This suggested limited multicollinearity.

### Sensitivity analyses

Full sensitivity analysis results are available from the authors on request. The findings from sensitivity analysis 2 (including all 96,577 trios with some genetic or phenotypic data) were comparable to the main findings. Some significant findings were not replicated in sensitivity analysis 1 (including a smaller subsample of 19,884 trios with genetic data for all three members). Specifically, in the trio SEM, the effects of children's PGS_edu_ on cognitively simulating parenting (which were significant in analysis 2 [β = 0.03, 95% CI [0.01, 0.05], *p* = .019] and the main analysis) were not statistically significant in analysis 1 (β = 0.02, 95% CI [−0.01, 0.05], *p* = .180). Similarly, the mediated effect of children's PGS_edu_ on their educational outcomes via literacy‐focused parenting (which were significant in analysis 2 [a*b: β = 0.01, 95% CI [1 × 10^−3^, 0.01], *p* = .018] and the main analysis) were not significant in analysis 1 (a*b: β = 4 × 10^−3^, 95% CI [−1 × 10^−3^, 0.01], *p* = .173). All other findings were similar across the three analyses.

## Discussion

We found mixed findings on the three study hypotheses: first, the results suggested evocative effects of children's education‐linked genetic propensities (PGS_edu_) on literacy‐focused (but not positive) maternal parenting; second, the positive associations between children's PGS_edu_ and their educational performance were significantly mediated via literacy‐focused (but not positive) maternal parenting; third, the association between children's PGS_edu_ and their educational outcomes was significantly mediated via their earlier language skills. Additionally, parent PGS_edu_ were unexpectedly negatively associated with positive and literacy‐focused maternal parenting, and children's language was positively associated with positive and literacy‐focused maternal parenting.

### Maternal parenting

We found evidence suggesting small statistically significant evocative effects of children's PGS_edu_ on literacy‐focused parenting, consistent with findings from a multivariate twin study (Tucker‐Drob & Harden, 2012) and analysis of PGS_edu_ in mother–child dyads (Wertz et al., 2020). We found no strong evidence of evocative effects on positive parenting, conflicting with results from Wertz et al. (2020) and Austerberry et al. (2024), the latter demonstrating positive associations between birth mother intellectual performance (a proxy for adoptee genetics) and the positive parenting of both adoptive parents.

The positive association between children's PGS_edu_ and their educational outcomes was significantly mediated via earlier literacy‐focused (but not positive) parenting. This cannot be directly compared with other findings as ours is the first study to test whether genetic effects on educational outcomes are mediated via literacy‐focused parenting. However, the lack of significant mediation via positive parenting is consistent with Austerberry et al. (2024), which demonstrated no significant mediation of genetic effects on educational outcomes via earlier positive parenting.

In line with previous research (Wertz et al., 2023), there were small statistically significant associations between mothers' PGS_edu_ and their parenting. However, contrary to results from the Dunedin Study, Environmental Risk longitudinal twin study, and Millennium Cohort Study, demonstrating parents with higher PGS_edu_ tended to be warmer and more sensitive and stimulating in their parenting (Wertz et al., 2023), we found that mothers with higher PGS_edu_ tended to self‐report less positive and literacy‐focused parenting. These associations run counter to theories that higher parental education may encourage positive parenting (Davis‐Kean, 2005; Davis‐Kean, Tang, & Waters, 2019) and evidence that better educated parents tend to spend more time with their children overall and engaging them in educationally promotive activities (Kalil, Ryan, & Corey, 2012; Suizzo & Stapleton, 2007), and tend to be more positively and emotionally responsive (Klebanov, Brooks‐Gunn, & Duncan, 1994). Consequently, it is unclear whether the negative associations between parent PGS_edu_ and maternal parenting represent a genuine association or a spurious one induced by, for example, collider bias, which occurs when sample selection invertedly controls for a variable (the ‘collider’) that is independently influenced by both the predictor and outcome, distorting the association between them.

However, it is also plausible that this association represents a true effect because, although unexpected, reverse negative findings are fairly common in this area. For example, parental PGS_edu_ were negatively associated with parental warmth in the Avon Longitudinal Study of Parents and Children (Wertz et al., 2023) and parental educational attainment was phenotypically negatively associated with maternal literacy‐focused parenting at 5 and 8 years in MoBa (Havdahl et al., 2023). A potential explanation for these negative effects is that parents with higher levels of education are busier and have less time to engage in positive parenting. Negative associations were also found between fathers' PGS_edu_ and positive maternal parenting, suggesting that fathers' genetics may evoke differences in their co‐parent's positivity, highlighting the importance of including fathers in parenting research.

The effects of parents' PGS_edu_ on children's educational performance were mostly mediated via children's PGS_edu_, suggesting the bivariate associations between parent PGS_edu_ and child educational performance are largely attributable to genetic transmission. Our incidental finding that direct associations between parent PGS_edu_ and children's educational outcomes were not significant offered little evidence of genetic nurture. This diverges from evidence of genetic nurture effects on education from several studies, primarily in older samples (B. Wang et al., 2021), including results from MoBa when the children were older (10–13 years) and using registry data on national exams (Isungset et al., 2021).

### Language

Children's PGS_edu_ were significantly associated with their language, and language significantly mediated the association between children's PGS_edu_ and their educational outcomes. This is consistent with findings from Austerberry, Fearon, et al. (2022) and Verhoef et al. (2021), reinforcing the theory that language may be an early manifestation of genetic effects on educational performance. Language and parenting were significantly positively associated at 5 years. As these associations were cross‐sectional, it is unclear whether they represent child‐to‐parent or parent‐to‐child effects. Research suggests both are plausible in language development (Lugo‐Gil & Tamis‐LeMonda, 2008; Tucker‐Drob & Harden, 2012). The associations between parent PGS_edu_ and child language were predominantly mediated via children's PGS_edu_, suggesting associations between parent PGS_edu_ and child language are mostly explained by genetic transmission. There were no significant unmediated effects of parent PGS_edu_ on child language; thus, there is no robust evidence of genetic nurture.

### Limitations and future directions

The findings should be interpreted considering several limitations. First, PGS currently explain a fraction of heritability (the optimistic upper bound for the proportion of phenotypic variance that can be explained by PGS). PGS_edu_ explained 11% of the variance in parent educational attainment in our sample, compared to twin heritability estimates of 40–56% (Branigan, McCallum, & Freese, 2013; Silventoinen et al., 2020; Wolfram & Morris, 2023). This discrepancy can be conceptualized as measurement error (Pingault et al., 2021; Tucker‐Drob, 2017). Consequently, PGS_edu_ effects (including estimates of assortative mating) were likely underestimated, and null effects may be false negatives. The low reliability of the PGS_edu_ also limited our control of passive *r*GE (i.e. genetic confounding) (Pingault et al., 2022), as did our analysis of biologically related parent‐offspring (making it difficult to separate genetic from shared environmental effects). Genetic confounding is more robustly ruled out by the children of twins (D'Onofrio et al., 2003) and parent‐offspring adoption designs, which more effectively isolate genetic and environmental effects. Underestimation of genetic effects can also have consequences for estimating the mediated effects of PGS_edu_ (Pingault et al., 2022). In mediation models, if there is an omitted variable influencing the mediator and outcome, the association between them will be exaggerated (Fritz, Kenny, & MacKinnon, 2016; Judd & Kenny, 1981). Thus, if the genetic propensity not captured by the children's PGS_edu_ is an unmeasured confounder influencing the mediator (language or parenting) and outcome (education), mediation will be overestimated.

Trio‐based analyses need large samples because of the strong associations between parent and child genotypes, leaving less independent variation to leverage when testing associations between the PGS_edu_ of each trio member and phenotypic outcomes. This may partly explain why our effect sizes were small, and some of the *p*‐values (e.g., for the association between the child PGS_edu_ and literacy‐focused parenting) were somewhat marginal.

The phenotypic measures were also somewhat limited. For example, as the parenting measures captured maternal parenting only, it is unclear whether our results generalize to the parenting of fathers, who are generally underrepresented in developmental research (Phares, Lopez, Fields, Kamboukos, & Duhig, 2005). We also relied on self‐reports, which are vulnerable to ceiling effects and rater bias, for example social desirability bias, which may partly account for the negative associations between parental PGS_edu_ and parenting if parents with lower PGS_edu_ are more susceptible to this bias. Phenotypic measurement presents a particular challenge for genomic research, relying on large sample sizes, as it is not usually possible to collect in‐depth measures at the scale required. As other MoBa analyses (e.g., Havdahl et al., 2023) have uncovered different results when analyzing national test scores instead of maternal reports of children's performance, future research is needed replicating the present analyses using national exam results.

MoBa is Norway‐based, and we only included participants whose genetic data indicated European ancestry, as the education GWAS used to construct the PGS_edu_ was conducted on European ancestry individuals. It is a serious limitation of GWASs that they have been conducted primarily in European ancestry populations (Peterson et al., 2019). PGS currently show poor generalizability in non‐European ancestry populations, and efforts are underway to increase their accuracy across diverse groups (Wang, Tsuo, Kanai, Neale, & Martin, 2022). Once possible, our results should be confirmed in different ancestral groups.

Norway is relatively affluent, with a high standard of living and high equality (The World Bank, 2023). Prior to the start of compulsory schooling at 6 years, most Norwegian children attend kindergarten. In 2004, when earliest‐recruited children reached 5 years old (the earliest timepoint analyzed), 88% of children in Norway aged 3–6 years were in full‐time kindergarten, rising to 97% by 2013, when last‐recruited children reached 5 years old (The Norwegian Directorate of Education, 2020). More equal educational environments plausibly increase the likelihood of genetic variation accounting for a higher proportion of variance in educational phenotypes (Asbury & Plomin, 2013; Scarr‐Salapatek, 1971). If so, the relatively uniform Norwegian childcare and schooling environment may result in stronger associations between children's PGS_edu_ and their language and educational outcomes, compared to countries with less equal early environments.

Attrition analysis suggests, as with other large cohort studies, participants who remained in the study were better educated and higher earning on average than those who did not respond to follow‐up or dropped out (Vejrup, Magnus, & Magnus, 2022). We cannot assume our findings generalize to families in low socioeconomic status (SES), particularly as parent SES appears to moderate genetic effects on children's cognitive and educational outcomes (Capron & Duyme, 1989; Tucker‐Drob & Bates, 2015; Turkheimer, Haley, Waldron, D'Onofrio, & Gottesman, 2003) and may causally influence parenting (Akee, Copeland, Keeler, Angold, & Costello, 2010; Cancian, Yang, & Slack, 2013). Future research should examine these associations in lower SES samples or apply probability weights, adjusting the contributions of individuals in the sample to better reflect the broader population.

## Conclusion

This is the first study to examine evocative effects on parenting of children's genetic propensity for education, while controlling for the genetics of both parents. Our findings suggest early language and literacy‐focused parenting may be important mechanisms in the pathway from genes to educational outcomes. This is informative for the development of promotive and preventative intervention and research into the causal mechanisms involved in the etiology of educational performance.

## Ethical consideration

The establishment of MoBa and initial data collection was based on a license from the Norwegian Data Protection Agency and approval from the Regional Committees for Medical and Health Research Ethics (REK). The MoBa cohort is based on the regulations of the Norwegian Health Registry Act. All participating mothers and fathers provided informed written consent. The current study was approved by the REK (2020, #21076).

**Key points**

- It has been hypothesized that increasing heritability of cognitive and educational abilities with age is partly due to evocative gene–environment correlation (*r*GE). However, this has not been robustly tested.
- Our study was the first to examine evocative *r*GE effects on educational performance, while controlling for the genetics of both parents. Our findings suggest: (a) children's education‐linked genetics may evoke differences in literacy‐focused maternal parenting, in turn influencing children's educational outcomes; (b) children's language mediates genetic effects on educational outcomes.
- Our results indicate language and parenting may be suitable targets for interventions promoting the development of educational skills and associated positive life outcomes. They also pave the way for research examining causal mechanisms implicated in the development of educational abilities.

## Supporting information


**Appendix S1.** Derivation of a subsample of unrelated trios.
**Appendix S2.** Construction of education polygenic scores.
**Appendix S3.** Exploratory factor analyses.
**Appendix S4.** Structural equation models.
**Appendix S5.** Missing data.
**Table S1.** Results from exploratory factor analysis of parenting items.
**Table S2.** Results from exploratory factor analysis of child language items.
**Table S3.** Results from exploratory factor analysis of child educational performance items.

## Data Availability

The data are managed by the Norwegian Institute of Public Health (NIPH) and can be made available to researchers with approval from the Regional Committees for Medical and Health Research Ethics (REK), in compliance with the EU GDPR and approval from the data owners. Participant consent does not permit individual‐level data storage in repositories or journals. Researchers wanting data access for replication should apply through helsedata.no. Data access requires approval from the REK and MoBa.
