# Supplementary material for: Evocative effects of children's education‐associated genetics on maternal parenting: results from the Norwegian mother, father and child cohort study
Source: J Child Psychol Psychiatry. 2025 Aug 4;67(2):158–70. doi: 10.1111/jcpp.70025 (PMC12812787; doi:10.1111/jcpp.70025)
Supplement: Supplementary file 1 — Appendix S1. Derivation of a subsample of unrelated trios. Appendix S2. Construction of education polygenic scores. Appendix S3. Exploratory factor analyses. Appendix S4. Structural equation models. Appendix S5. Missing data. Table S1. Results from exploratory factor analysis of parenting items. Table S2. Results from exploratory factor analysis of child language items. Table S3. Results from exploratory factor analysis of child educational performance items. [file JCPP-67-158-s001.docx]

**Supporting Information**

**Appendix S1.** Derivation of a Subsample of Unrelated Trios.

For the analyses in this study, we used a subsample of unrelated parent-offspring trios with post-QC genetic data available for at least one trio member. Due to extensive relatedness among parents in MoBa, we performed post-imputation quality control steps, in addition to those by Corfield et al. (2022), to ensure the trios included in our analyses were indeed unrelated. Further, because the genotypes in MoBa were obtained and imputed in multiple batches (Corfield et al., 2022), we also incorporated steps to minimize batch effects.

To be certain that only independent (unrelated) trios were included in the analyses, we addressed the complex relatedness structure between MoBa participants by removing one individual from each pair of individuals (not belonging to a trio) who shared more than 15% relatedness, measured as PI_HAT in PLINK 1.9 (Purcell et al., 2007), prioritizing the inclusion of children, then mothers and then fathers. Within families with siblings, one (or more) child was randomly removed to create a trio.

As the data were genotyped and imputed in multiple batches, we removed single nucleotide polymorphisms (SNPs) that were significantly different in their minor allele frequencies (MAFs) between batches (*p* < 1.00 x 10**^−^**^03^). To further address any possible technical artifacts, we performed principal components analysis (PCA) in PLINK 1.9 (Purcell et al., 2007) in the subsample selected for the presented analyses.

**Appendix S2.** Construction of Education Polygenic Scores.

First, we identified SNPs present in both the summary statistics (not including 23andMe) from the Okbay et al. (2022) genome-wide association study (GWAS) of years of education (hereafter referred to as EA4) and the European ancestry Linkage Disequilibrium (LD) reference panel constructed using the 1000 Genomes Project phase 3 samples (McVean et al., 2012). Second, we subjected these SNPs to quality control (QC) steps outlined in Choi, Mak, and O’Reilly (2020). Specifically, we checked and, if identified, removed variants that: were not SNPs, were strand ambiguous, had imputation quality (INFO score) below 90%, had MAF below 1% in the summary statistics of Okbay et al. (2022) or the reference panel, had MAF difference between EA4 and the reference panel greater than 20%, had mismatching alleles between EA4 and the reference panel, had out of bounds *p*-values (1.00 < p < .00), and had duplicated IDs. Third, we used PRS-CS to shrink the effect sizes of SNPs present in EA4 and the LD reference panel. To do so, we used the following PRS-CS parameters: 25,000 Markov chain Monte Carlo (MCMC) iterations, 10,000 burnin iterations, global shrinkage factor = .01, parameter a in the gamma-gamma prior = 1, parameter b in the gamma-gamma prior = .5, MCMC thinning factor = 5. Fourth, we subjected the SNPs in the MoBa subsample selected for our analyses to the same QC steps as the EA4 SNPs described above. Finally, the polygenic scores (PGS_edu_)were calculated in PLINK 1.9 (Purcell et al., 2007), using the SNP effect sizes processed by PRS-CS. Overall, 502,729 SNPs passed the QC and were present in the summary statistics, MoBa data and the LD reference panel. These SNPs were used to calculate the PGS_edu_ for 161,566 individuals (63,032 children, 58,778 mothers, 39,756 fathers).

Prior to hypothesis testing, the PGS_edu_ were standardized to have a mean of 0 and variance of 1. Residualized PGS_edu_ were then calculated by regressing the standardized PGS_edu_ on sex, genotyping plate (to control for plate effects, which have been detected in the MoBa sample) and the first 5 principal components derived from a principal component analysis (PCA) of the MoBa subsample created for these analyses (without the 1000 Genomes reference). To test the performance of the residualized PGS_edu_, prior to the main analyses, we regressed the parent PGS_edu_ on measures of parent educational attainment. Maternal PGS_edu_ were significantly (*p* < .001) associated with maternal education (captured using a self-report item on educational attainment included in the questionnaire sent to mothers at 15 weeks of pregnancy) and explained 10.52% of the variance in maternal education. Similarly, paternal PGS_edu_ were significantly (*p* < .001) associated with paternal education (measured using a self-report item on level of education included in the questionnaire sent to fathers at 15 weeks of pregnancy) and explained 11.28% of the variance in paternal education. The associations between the PGS_edu_ and parent educational attainment were in the expected (positive) direction. The variance explained was comparable to (but slightly smaller than) the 12–16% of educational attainment variance explained by the PGS_edu_ created by Okbay et al. (2022), based on the same GWAS we used to construct our PGS_edu_ (although they used additional GWAS data from 23andMe, not included in our analyses, so the results are not directly comparable). As a further test of the performance of the PGS_edu_, we regressed the residualized mother, father, and child education PGS_edu_ on a negative control (child height, reported by mothers in response to the questionnaire sent to them when their child was 8 years of age). As expected, the PGS_edu_ were not significantly (*p* > .05) associated with child height and explained less than 0.00% of the variance in child height.

**Appendix S3.** Exploratory Factor Analyses.

Prior to hypothesis testing, to determine which items to include in the latent variables constructed for the main analysis, we conducted the following three exploratory factor analyses (EFAs) of data on a subsample of 28,818 children who had been excluded from the main analysis (because genetic data were not available for them or either of their parents), 12,950 of whom had some of the relevant phenotypic data available.

**Maternal Parenting.** We examined the factorability of 11 parenting items from the questionnaire sent to mothers when children were 5 years of age. Two of the 11 items were on literacy-focused parenting (Prior, Bavin, & Ong, 2011) (see the measures section of the main manuscript) and nine were from the Alabama Parenting Questionnaire (APQ) (Frick, 1991). Of the nine APQ items, six were from the ‘Positive Parenting’ subscale (see the measures section of the main manuscript) and three were from the ‘Consistency of the Use of Discipline’ subscale. The three items on ‘Consistency of the Use of Discipline’ were answered on a 5-point scale from ‘1-Never’ to ‘5-Always’ and reverse scored ahead of the EFA, so they were in the same direction as the other parenting items (with higher scores indicating more literacy-focused, positive, consistent parenting). A correlation matrix of the 11 parenting items revealed that all items correlated > .30 with at least one other item, suggesting reasonable factorability. We used version 2.0.12 of the R package psych (Revelle, 2020) to conduct Bartlett’s Test of Sphericity, which tests whether variables are suitable for data reduction techniques such as EFA by comparing the observed correlation matrix to the identity matrix. The Bartlett’s Test result was statistically significant, suggesting the parenting variables were suitable for EFA: χ^2^(55) = 80,064.47, *p* < .001. To determine how many factors to extract, we created a scree plot of successive eigenvalues from a PCA of the 11 parenting items, using version 2.0.12 of the psych package in R (Revelle, 2020). Two widely used decision rules are to retain the factors to the left of the point of inflection in a scree plot of eigenvalues (Cattell, 1966) or to retain all factors with eigenvalues greater than 1 (Kaiser, 1960). In the scree plot of parenting items, the point of inflection was at component 4 and three eigenvalues were greater than 1. Both results indicated a three-factor solution. Using the R packages psych (version 2.0.12) (Revelle, 2020) and GPArotation (Bernaards & Jennrich, 2005), we ran a three-factor maximum likelihood factor analysis with oblique (oblimin) rotation (as we were expecting the factors to correlate). The results from the EFA are displayed in Table S1. Inspection of the items with the highest loadings on each factor suggested factor 1 represented positive parenting, factor 2 represented literacy-focused parenting, and factor 3 represented consistency in the use of discipline. Together, the three factors explained 45% of the total variance. Individually, they explained 21%, 13% and 11% of the variance, respectively. Factor 1 was weakly correlated with Factors 2 and 3, *r* = .26 and .14, respectively. Factors 2 and 3 were very weakly correlated, *r* = .06. The reliability of the first two factors was acceptable/good (positive parenting, α = .79; literacy-focused parenting, α = .81) and the reliability of the third factor, consistency in the use of discipline, was questionable (α = .66). We decided to retain Factors 1 and 2 (positive and literacy-focused parenting) in the main analysis as previous literature examining evocative *r*GE in cognitive and educational development (using different methods to ours) found significant associations between children’s education/cognition-associated genes and the positive and literacy-focused parenting they received (Austerberry et al., 2024; Tucker-Drob & Harden, 2012; Wertz et al., 2020).

**Child Language.** We examined the factorability of 41 items measuring children’s language at 5 years of age. Six of the 41 items were from the Coherence subscale of the Children’s Communication Checklist-2 (CCC-2), which is designed to assess the communication skills of children aged 4 to 16 years (Bishop, 2003, 2006) and was scored on a 4-point Likert scale from ‘1-Rarely or never’ to ‘4-Often or always’. Thirteen of the 41 items were from the Speech and Language Assessment Scale (SLAS) (Rice, Wilcox, Liebhaber, & Hadley, 1989) (see the measures section of the main manuscript). Twenty-two of the 41 items were on language-related difficulties, 20 of which were from the Checklist of 20 Statements about Language-Related Difficulties (Språk 20) (Ottem, 2009) and two of which were MoBa-specific questions. The former consists of 20 statements describing language-related difficulties, which can be further divided into three subscales: Semantics (eight items), Receptive (six items) and Expressive Language (six items). The two additional MoBa-specific questions asked how well the following statements fit the child: (1) ‘Gets tired quickly in tasks demanding attention to language’ and (2) ‘Avoids talking to people other than family members’. All 22 items on language related difficulties were scored on a 5-point Likert scale from ‘1-Doesn’t fit the child, absolutely wrong’ to ‘5-fits fine with the child, absolutely right’. A correlation matrix of the 41 language items revealed that the six items from the CCC-2 were weakly correlated with other items. The remaining 35 items correlated at least .30 with at least one other item, suggesting reasonable factorability. Bartlett’s Test of Sphericity was significant, suggesting the 35 items were suitable for EFA: χ^2^(595) = 766,258.10, *p* < .001. In a scree plot of successive eigenvalues, the point of inflection was at component 3 and five eigenvalues were greater than 1, suggesting either a two- or a five-factor solution. We ran a two-factor maximum likelihood factor analysis with oblimin rotation. The results from the EFA are displayed in Table S2. Inspection of the items with the highest loadings on each factor suggested factor 1 represented language difficulties and factor 2 represented general speech and language performance. Together, the two factors explained 53% of the total variance. Individually, they explained 28% (language difficulties) and 25% (speech and language) of the variance. The two factors were moderately correlated, *r* = .43. The reliability of each factor was excellent (language difficulties, α = .94; speech and language, α = .96). We decided to retain factor 2 (speech and language) in the main analysis, as our aim was to examine the role of normative language development rather than language impairments/difficulties.

**Child Educational Performance.** We examined the factorability of three items on educational performance. The items were from the questionnaire sent to mothers when the children were 8 years of age and asked about teacher feedback during parent-teacher discussions about the child’s performance in national exams on: (1) ‘… Reading skills in 1^st^ grade’, (2) ‘Reading skills in 2^nd^ grade’, (3) ‘Arithmetic skills in 2^nd^ grade’. Each item was scored on a 3-point Likert scale: ‘1-Has mastered subject well’, ‘2-Must work more but teacher is not concerned’ and ‘3-Teacher is concerned’. The three items were moderately to highly correlated (*r* range: .43–.76). Bartlett’s Test of Sphericity was significant, suggesting the three items were suitable for EFA: χ^2^(3) = 32,219.22, *p* < .001. In a scree plot of successive eigenvalues, the point of inflection was at component 2 and one eigenvalue was greater than 1, both suggesting a one-factor solution. We ran a one-factor maximum likelihood factor analysis. The results from the EFA are displayed in Table S3. The single factor explained 59% of the total variance and was retained for the main analysis. The reliability of the factor was acceptable (α = .78).

**Appendix S4.** Structural Equation Models.

We constructed the structural equation models (SEMs) in several steps: First, we ran bivariate SEMs, examining the associations between the PGS_edu_ (child, mother, and father, separately) and the four phenotypic variables: (1) the latent variable measuring child educational performance at 6–8 years of age, (2) the latent variable measuring child language at 5 years of age, (3) the latent variable measuring maternal positive parenting when children were 5 years of age, (4) the observed variable measuring maternal literacy-focused parenting when children were 5 years of age. Second, we ran four trio SEMs, which jointly modelled the effects of child, mother, and father PGS_edu_ on the four phenotypic variables. The two trio SEMs examining the effects of the trio PGS_edu_ on maternal parenting at 5 years old were a test of study hypothesis 1: there would be evocative effects of children’s education PGS_edu_ on parenting, after controlling for parent PGS_edu_. Third, we examined whether the effect of children’s education-linked genetic propensities (PGS_edu_) on their educational performance at 6–8 years would be mediated via parenting at 5 years (study hypothesis 2), in two models (one examining mediation via positive maternal parenting and the second testing for mediation via literacy-focused maternal parenting). Specifically, we combined each of the two trio SEMs examining the effects of the trio PGS_edu_ on parenting with the trio SEM examining the effects of the trio PGS_edu_ on educational performance at 6–8 years old and calculated the mediated effect of children’s PGS_edu_ on their educational performance via maternal parenting. Fourth, we examined whether the effect of children’s PGS_edu_ on their educational performance at 6–8 years of age was mediated via their language at 5 years old (study hypothesis 3). Specifically, we combined the trio SEM examining the effects of the trio PGS_edu_ on language with each of the two models (one on positive maternal parenting and the second on literacy-focused maternal parenting) constructed in the aforementioned third step and calculated the mediated effect of children’s PGS_edu_ on their educational performance at 6–8 years via their language performance at 5 years. We also included a path in the model, examining the association between maternal parenting at 5 years and children’s language at 5 years.

**Appendix S5.** Missing Data.

Missing data were handled using full information maximum likelihood (FIML), which simulation studies suggest outperforms listwise deletion and produces unbiased parameter estimates and standard errors when the data are missing at random (MAR) or missing completely at random (MCAR) (Enders & Bandalos, 2001). The data used in the study were not missing completely at random (MCAR) according to results from the Little's MCAR test, conducted in the naniar package version 0.6.1 (Tierney, Cook, McBain, & Fay, 2021) in R version 4.0.3 (R Core Team, 2020): χ^2^ (9,302) = 11,543, *p* < .001. MCAR occurs when the probability of being missing is the same for all cases and there is no systematic association between the missingness of the data and any other values, observed or missing. As the data were not MCAR, we ran an additional attrition analysis using the Missing Value Analysis function in IBM SPSS Statistics for Windows version 28.0, which creates an indicator variable identifying variables that contain missing values. This indicator value was used to compare group means among different variables in the data set, using the *t*-test procedure. In the attrition analysis, the patterns of missingness for all study variables were related to the observed values of one or more other variables in the data set, ruling out the possibility that the data were MCAR. It was not possible to rule out the possibility that the data were missing not at random (MNAR), which is when the missingness of the data is systematically related to unobserved data. However, the results from the attrition analysis were consistent with the data being missing at random (MAR), which occurs when the missingness of a variable is systematically related to the observed but not the unobserved data.

**Table S1**. Results from Exploratory Factor Analysis of Parenting Items.

|  | Oblimin rotated factor loadings | | | |
| --- | --- | --- | --- | --- |
| Item | Positive | Literacy-  Focused | Consistency in discipline |  |
| You let your child know when he/she is doing a good job | **0.59** | 0.01 | 0.08 |  |
| You have a friendly talk with your child | **0.39** | 0.09 | 0.09 |  |
| You ask your child about his/her day in childcare | **0.47** | 0.03 | 0.03 |  |
| You compliment your child when he/she has done something well | **0.80** | −0.04 | −0.01 |  |
| You praise your child if he/she behaves well | **0.79** | 0.01 | −0.03 |  |
| You talk to your child about his/her friends | **0.58** | 0.09 | −0.03 |  |
| You threaten to punish your child and then do not actually punish him/her (reverse scored) | 0.04 | 0.03 | **0.68** |  |
| Your child talks him/herself out of being punished after he/she has done something wrong (reverse scored) | 0.01 | −0.02 | **0.56** |  |
| You let your child out of a punishment early (e.g. lift restrictions earlier than you originally said) (reverse scored) | −0.05 | −0.02 | **0.66** |  |
| During a typical week, how often to you teach your child how to print letters and words? | 0.00 | **0.88** | −0.02 |  |
| During a typical week, how often to you help your child read letters and sounds? | 0.00 | **0.78** | 0.02 |  |
| Eigenvalues | 2.35 | 1.42 | 1.24 |  |
| Proportion of variance | .21 | .13 | .11 |  |

*Note.* Factor loadings over 0.40 appear in bold.

**Table S2.** Results from Exploratory Factor Analysis of Child Language Items.

|  | Oblimin rotated factor loadings | |
| --- | --- | --- |
| Item | Language difficulties | Speech and language |
| Forgets words s/he knows the meaning of (reverse scored) | **0.56** | 0.03 |
| Confuses words with similar meaning (reverse scored) | **0.54** | 0.00 |
| Has difficulty understanding the meaning of common words (reverse scored) | **0.65** | −0.05 |
| Has difficulty answering questions as quickly as other children (reverse scored) | **0.71** | 0.04 |
| Is often searching for the right words (reverse scored) | **0.65** | 0.02 |
| Uses incomplete sentences (reverse scored) | **0.68** | 0.06 |
| Uses short sentences when s/he answers questions (reverse scored) | **0.56** | 0.04 |
| Has difficulty retelling a story s/he has heard (reverse scored) | **0.69** | 0.08 |
| Is quickly getting tired in tasks demanding attention to language (reverse scored) | **0.77** | 0.02 |
| It doesn't seem like what s/he is learning is remembered (reverse scored) | **0.75** | −0.05 |
| Has difficulty remembering things (reverse scored) | **0.69** | −0.05 |
| Has difficulty understanding what others are saying (reverse scored) | **0.78** | −0.06 |
| Misconceive instructions and messages (reverse scored) | **0.76** | −0.06 |
| Has problems remembering messages (reverse scored) | **0.68** | −0.05 |
| Misunderstands context and what is going on (reverse scored) | **0.74** | −0.05 |
| Is difficult to understand (reverse scored) | **0.69** | 0.06 |
| Has difficulty expressing wishes and needs (reverse scored) | **0.68** | 0.01 |
| Is not understood by others (reverse scored) | **0.69** | 0.07 |
| Seldom initiates conversations with others (reverse scored) | **0.51** | −0.02 |
| Has difficulties in pronunciation (reverse scored) | **0.55** | 0.16 |
| Is not able to have a dialogue with peers (reverse scored) | **0.70** | 0.02 |
| Avoids talking to other than family members (reverse scored) | **0.46** | −0.02 |
| My child's ability to ask question properly is… | 0.01 | **0.82** |
| My child's ability to answer question properly is… | 0.03 | **0.82** |
| My child's ability to say sentences clearly enough to be understood by strangers is… | 0.04 | **0.82** |
| The number of words my child knows is… | −0.04 | **0.83** |
| My child's ability to use his/her words correctly is… | −0.02 | **0.87** |
| My child's ability to get his/her message across to others when talking is… | 0.00 | **0.88** |
| My child's ability to use proper words when talking to others is | 0.00 | **0.88** |
| My child's ability to get what he/she wants by talking is… | −0.03 | **0.83** |
| My child's ability to start a conversation going with other children is... | −0.04 | **0.72** |
| My child's ability to keep a conversation going with other children is... | −0.01 | **0.73** |
| The length of my child's sentences is… | −0.01 | **0.86** |
| My child's ability to make 'grown up' sentences is… | 0.02 | **0.80** |
| My child's ability to correctly say the sounds in individual words is… | 0.08 | **0.68** |
| Eigenvalues | 9.77 | 8.73 |
| Proportion of variance | .28 | .25 |

*Note.* Factor loadings over 0.40 appear in bold

**Table S3**. Results from Exploratory Factor Analysis of Child Educational Performance Items.

| Item | Factor loadings |
| --- | --- |
| What feedback have you received about your child's reading skills in 1st Grade | **0.84** |
| What feedback have you received about your child's reading skills in 2nd Grade | **0.92** |
| What feedback have you received about your child's arithmetic skills in 1st Grade | **0.48** |
| Eigenvalue | 1.77 |
| Proportion of variance | .59 |

*Note.* Factor loadings over 0.40 appear in bold.

**References**

Austerberry, C., Fearon, P., Ronald, A., Leve, L. D., Ganiban, J. M., Natsuaki, M. N., . . . Reiss, D. (2024). Evocative effects on the early caregiving environment of genetic factors underlying the development of intellectual and academic ability. *Child Development, 00*, 1–20. doi:10.1111/cdev.14142

Bernaards, C. A., & Jennrich, R. I. (2005). Gradient projection algorithms and software for arbitrary rotation criteria in factor analysis. *Educational and Psychological Measurement, 65*(5), 676-696. doi:10.1177/0013164404272507

Cattell, R. B. (1966). The scree test for the number of factors. *Multivariate Behavioral Research, 1*(2), 245-276.

Choi, S. W., Mak, T. S.-H., & O’Reilly, P. F. (2020). Tutorial: A guide to performing polygenic risk score analyses. *Nature Protocols, 15*(9), 2759-2772. doi:10.1038/s41596-020-0353-1

Corfield, E., Frei, O., Shadrin, A. A., Rahman, Z., Lin, A., Athanasiu, L., . . . Havdahl, A. (2022). The Norwegian Mother, Father, and Child cohort study (MoBa) genotyping data resource: MoBaPsychGen pipeline v.1. Preprint: <https://www.biorxiv.org/content/10.1101/2022.06.23.496289v2>.

Enders, C. K., & Bandalos, D. L. (2001). The relative performance of full information maximum likelihood estimation for missing data in structural equation models. *Structural Equation Modeling: A Multidisciplinary Journal, 8*(3), 430-457. doi:10.1207/S15328007SEM0803_5

Frick, P. J. (1991). *Alabama Parenting Questionnaire*. Unpublished rating scale: University of Alabama.

Kaiser, H. F. (1960). The application of electronic computers to factor analysis. *Educational and Psychological Measurement, 20*(1), 141-151.

McVean, G. A., Altshuler, D. M., Durbin, R. M., Abecasis, G. R., Bentley, D. R., Chakravarti, A., . . . Ruiz-Linares, A. (2012). An integrated map of genetic variation from 1,092 human genomes. *Nature, 491*(7422), 56-65. doi:10.1038/nature11632

Okbay, A., Wu, Y., Wang, N., Jayashankar, H., Bennett, M., Nehzati, S. M., . . . LifeLines Cohort, S. (2022). Polygenic prediction of educational attainment within and between families from genome-wide association analyses in 3 million individuals. *Nature Genetics, 54*(4), 437-449. doi:10.1038/s41588-022-01016-z

Ottem, E. (2009). 20 spørsmål om språkferdigheter – en analyse av sammenhengen mellom observasjonsdata og testdata. *Skolepsykologi, 1*, 11–27.

Prior, M., Bavin, E., & Ong, B. (2011). Predictors of school readiness in five‐ to six‐year‐old children from an Australian longitudinal community sample. *Educational Psychology, 31*(1), 3-16. doi:10.1080/01443410.2010.541048

Purcell, S., Neale, B., Todd-Brown, K., Thomas, L., Ferreira, M. A., Bender, D., . . . Sham, P. C. (2007). PLINK: A tool set for whole-genome association and population-based linkage analyses. *American Journal of Human Genetics, 81*(3), 559-575. doi:10.1086/519795

R Core Team. (2020). *A language and environment for statistical computing*. Vienna, Austria: R Foundation for Statistical Computing.

Revelle, W. (2020). *psych: Procedures for personality and psychological research*. Illinois, USA: Northwestern University.

Rice, M. L., Wilcox, K. A., Liebhaber, G. K., & Hadley, P. A. (1989). *The speech and Language Assessment Scale*. Unpublished: University of Kansas, USA.

Tierney, N., Cook, D., McBain, M., & Fay, C. (2021). *naniar: Data structures, summaries, and visualisations for missing data. R package version 0.6.1.*

Tucker-Drob, E. M., & Harden, K. P. (2012). Early childhood cognitive development and parental cognitive stimulation: Evidence for reciprocal gene–environment transactions. *Developmental Science, 15*(2), 250-259. doi:10.1111/j.1467-7687.2011.01121.x

Wertz, J., Moffitt, T. E., Agnew-Blais, J., Arseneault, L., Belsky, D. W., Corcoran, D. L., . . . Caspi, A. (2020). Using DNA from mothers and children to study parental investment in children's educational attainment. *Child Development, 91*(5), 1745-1761. doi:10.1111/cdev.13329
